# Surgery for facial palsy in the hands of otorhinolaryngologists: a population-based study

**DOI:** 10.1007/s00405-024-09044-7

**Published:** 2024-10-23

**Authors:** Elisabeth Alberts, Jonas Ballmaier, Daniel Boeger, Jens Buentzel, Kerstin Hoffmann, Jiří Podzimek, Holger Kaftan, Andreas Mueller, Sylvia Tresselt, Gerd Fabian Volk, Orlando Guntinas-Lichius

**Affiliations:** 1 Department of Otorhinolaryngology, Jena University Hospital, Jena, Germany; 2 Department of Otorhinolaryngology, Zentralklinikum, Suhl, Germany; 3 Department of Otorhinolaryngology, Südharz-Krankenhaus gGmbH, Nordhausen, Germany; 4 Department of Otorhinolaryngology, Sophien/Hufeland-Klinikum, Weimar, Germany; 5 Department of Otorhinolaryngology, Klinikum Bad Salzungen, Bad Salzungen, Germany; 6 Department of Otorhinolaryngology, Helios-Klinikum, Erfurt, Germany; 7 Department of Otorhinolaryngology, SRH Wald-Klinikum, Gera, Germany; 8 Department of Otorhinolaryngology, Ilm-Kreis-Kliniken, Arnstadt, Germany; 9 Facial-Nerve-Center, Jena University Hospital, Jena, Germany; 10 Center for Rare diseases, Jena University Hospital, Jena, Germany

**Keywords:** Facial nerve, Facial palsy, Facial paralysis, Reconstructive surgery, Facial reanimation, Healthcare research

## Abstract

**Purpose:**

Modern facial surgery can improve eye closure and address facial functional and emotional expression disabilities in case of severe acute facial paralysis with low probability of recovery and in cases of chronic flaccid facial paralysis. Reports on outcome typically originate from specialized tertiary care centers, whereas population-based data from routine care beyond specialized centers is sparse.

**Methods:**

Therefore, patients’ characteristics, surgical techniques, postoperative complications, and patients’ satisfaction with the final outcome were analyzed for all inpatients with facial paralysis undergoing facial surgery in Thuringia, a federal state in Germany, between 2006 and 2022. 260 patients (female 41.5%; median age 65 years) were included.

**Results:**

On average, the surgery rate was higher for men than for women (0.83 ± 0.39 versus 0.58 ± 0.24 per 100,000 population per year). For first surgery, static procedures were dominating (67.3%), followed by dynamic reconstruction (13.8%), and combined static and dynamic reconstructions (13.5%). The most frequent type of surgery was upper lid weight loading (38.5%), hypoglossal-facial jump nerve suture (17.3%), and facial-facial interpositional graft suture (16.9%). Bleeding/hematoma formation needing revision surgery was the most frequent complication (6.2%). Overall, 70.4% of the patients were satisfied with the final result. The satisfaction was higher if the target was to improve eye closure (65.2%) or to improve upper face function (65.3%) than to improve the lower face function (53.3%).

**Conclusions:**

If facial nerve reconstruction surgery and/or upper lid weight placement was performed, the satisfaction was significantly higher. If revision surgery was needed to improve the result, the satisfaction was significantly lower.

**Supplementary Information:**

The online version contains supplementary material available at 10.1007/s00405-024-09044-7.

## Introduction

Peripheral facial palsy is the most common cranial nerve disorder. The incidence is estimated with about 7–40 patients/100,000 population per year [1]. The lesion is mainly mild in idiopathic facial nerve palsy, being by far the most common form. Therefore, full recovery occurs in about 75% of these cases [1, 2]. Severe facial nerve lesions like in tumor or trauma cases mostly lead to incomplete recovery, recovery with defect healing, or failure to recover. Typical cases of severe facial nerve lesion without the possibility of spontaneous recovery are, for example, patients with tumor infiltration of the extratemporal nerve portion and removal during surgery of a malignant parotid tumor, or patients with transection of the nerve at the cerebellopontine angle during surgery for a vestibular schwannoma [3, 4]. Due to the lack of regeneration of facial nerve fibers, the acute facial paralysis progresses to a chronic flaccid facial paralysis if no facial nerve reconstruction is performed. This leads to drinking and eating impairments, poorer pronunciation, limited vision in the affected eye, and importantly, lack of emotional expression and limitations in nonverbal communication [5, 6]. All this and the stigmatization lead to a significantly reduced quality of life, psychosocial withdrawal and often also to depression in the affected persons [7].

Major indications for dynamic or static facial surgery are acute or chronic facial paralysis without probability of spontaneous recovery, improvement of facial function during the course of facial nerve regeneration, or treatment of postparalytic synkinesis [8]. A variety of surgical procedures have been established internationally in recent decades. Individual selection of the best combination of surgical techniques is necessary to achieve the best result for the individual patient often with participation of several surgical disciplines [9]. Otolaryngology/head and neck surgery is an important partner in this interaction.

Most data on surgery for facial paralysis is published from specialized facial nerve centers [10–13]. Population-based data reflecting surgery in the breadth of patient care beyond the specialized centers are sparse. Recently, a nationwide analysis of inpatient hospital healthcare data in Germany showed an increase of inpatient surgery rates in case of facial paralysis between 2005 and 2019, mainly for facial nerve reconstruction surgery and static sling surgery. Most surgeries were provided by otolaryngology/head and neck surgery (39%) and ophthalmology or dentistry, oral and maxillofacial surgery (20% each) [9].

To allow for a population-based approach for analyses of inpatient treatments in otolaryngology, head and neck surgery, all eight Department of Otorhinolaryngology, Head and Neck Surgery in Thuringia, a federal state in Germany with about two million inhabitants, have established a network pooling data of all inpatients treated in Thuringia for a selected disease. Using this structure, all patients with facial paralysis receiving facial surgery between 2006 and 2022 in the entire population of Thuringia were analyzed. This allowed a comprehensive analysis of the indications, used techniques, and outcome in a clinical-routine setting beyond clinical trials and specialized cancer centers.

## Methods

### Study design and setting

The institutional ethics committee of the Jena University Hospital, Jena, Germany, approved the study protocol for a retrospective data collection (no. 2726-12/09; no. 4370-03/15). The study followed the ethical standards outlined in the Declaration of Helsinki and was carried out in accordance with all other relevant guidelines and regulations. Only anonymized data were analyzed. Therefore, the ethics committee waived the need for written informed consent.

A standardized retrospective analysis was performed in all eight Thuringian hospitals with a department of otolaryngology (in alphabetic order: Arnstadt, Bad Salzungen, Erfurt, Gera, Jena, Nordhausen, Suhl, Weimar). The patients were selected who were coded with a facial palsy (G51.-, P11.3, G83.6 and Q07 due to the International Classification of Diseases [ICD], 10th revision, German modification; ICD-10-GM) and had surgery during the same treatment. All procedures related to surgery for facial palsy were recorded following the according to the operation and procedure codes (Operations- und Prozedurenschlüssel; OPS): 5-984, 5 − 04 (including 5–040 to 5–042, 5–044 to 5–049 and 5-04b), 5–05 (including 5–058.0 to 5-058.2, 5-058.4 and 5-058.5), 5-858 (including 5-858.70), 5–09 (including 5–092, 5–096 including 5-096.3, 5–097 and 5–099 including 5–099.0) and 5-910. In addition, the surgeries were classified in dynamic reconstructions (nerve reconstruction, muscle transfer), static reconstructions (any eyelid surgery, sling plasties), or combinations of both. Patients undergoing surgery to treat the facial palsy or sequelae between 2006 and 2022 were included. The analysis revealed that two of the eight department of otolaryngology (Arnstadt, Weimar) did not perform surgery for facial palsy.

A retrospective search of the patients’ charts was performed. The following variables were obtained: age, sex, comorbidity, medical treatment and surgical procedures to treat the facial palsy. During follow-up, many patients underwent further surgeries. All these surgeries for facial reanimation were also recorded. The Charlson comorbidity index (CCI) was used to measure the general comorbidity of the patients [14].

If a facial grading was performed preoperatively, it was done by the House-Brackmann grading scale and/or by the Stennert index [15, 16] The House-Brackmann grading scale is a six step scale from grade I (normal function) to grade VI (complete paralysis). The Stennert index is a double-weighted system and popular in Germany. The observer judges facial symmetry at rest in four categories (0 = normal resting tone/symmetry up to 4 = no resting tone/gross asymmetry) and the motility of the facial muscles in six categories (0 = normal motility up to 6 = complete paralysis). An initial screening of the patients’ charts showed that a facial grading was not used regularly to assess also the facial function after surgery. Furthermore, the aims of surgery were highly variable. Therefore, the patient’s satisfaction with the outcome of surgery was classified based on the information in the patients’ charts as follows: Overall improvement (yes/no), improved eye closure (yes/no), improved upper face (yes/no), improved lower face (yes/no).

The medical records were thoroughly examined to register each deviation of the postoperative course for each surgery. Every deviation from the standard treatment or the expected postoperative course was documented and regarded as a complication. Then, the management of the complication was recorded and used to grade according to the surgical Clavien-Dindo Classification (CDC) [17].

### Statistical analysis

Participants’ characteristics and outcome variables were analyzed with IBM SPSS statistics software (Version 28.0.0.0) for medical statistics. Data are presented as mean ± standard deviation (SD) if not otherwise indicated. The chi-square test was used to compare nominal data of two independent subgroups. Fisher’s exact test was used to compare ordinal data of ≥ 2 independent subgroups. The Mann-Whitney U-test was used to compare scaled data of two independent subgroups. In general, nominal p values of two-tailed tests are reported.

The epidemiological calculations for the incidence of surgery (surgical rate) for facial palsy per 100,000 population were based on the annual mean number of habitants in Thuringia from 2006 to 2022. Population numbers of the online database of the Thuringian State Office for Statistics (www.tls.thueringen.de) were used.

## Results

### Study participants

Patients’ characteristics and characteristics of the facial palsy are summarized in Tables 1 and 2. Two-hundred and sixty (260) patients (female 41.5%) were included. The median age at the time of the first surgery was 65 years. Comorbidity was predominately mild (CCI 1–2; 43.5%) or moderate (CCI 3–4; 22.7%). Nearly all palsies were the results of peripheral facial nerve lesions (97.7%). The predominant etiology was a trauma/post-surgical lesion (62.7%) followed by tumor infiltration (17.3%). 59.7% had a conservative treatment before presenting for surgery. The median interval between onset of the palsy and first surgery was 8.5 months (range: 0-843). Most patients underwent surgery in the chronic stage (> 4 months after onset; 66.2%) of the palsy. A facial grading was performed in 53.5% of the patients (House-Brackmann range: II-VI; Stennert index at rest range: 0–4; Stennert index in motion range: 1–6). The dominant facial function impairments leading to facial surgery are summarized in Supplement Table 1. The primary impairments in descending order were: upper face impaired (96.2%), eye closure impaired (86.5%), and lower face impaired (75.4%).

**Table 1:** Patients’ characteristics

Parameter	*n*	%
All patients	260	100
Gender		
Female	108	41.5
Male	152	58.5
Charlson Comorbidity Index		
CCI 0	32	12.3
CCI 1–2 (mild)	113	43.5
CCI 3–4 (moderate)	59	22.7
CCI ≥ 5 (severe)	56	21.5
	**Mean ± SD**	**Median**,** Range**
Age at onset of palsy, years	55.8 ± 21.5	60, 0–92
Age at first consultation, years	60 ± 19.4	63, 0–92
Age at first surgery, years	61 ± 18.9	65, 2–92
Charlson Comorbidity Index	3.35 ± 2.92	2, 0–13

**Table 2:** Characteristics of the facial palsy

Parameter	*n*	%
All patients	260	100
Side		
Left	141	54.2
Right	112	43.1
Bilateral	7	2.7
Lesion site		
Central	3	1.2
Peripheral	254	97.7
Nuclear	3	1.2
Onset at birth		
Yes	9	3.5
No	251	96.5
Etiology		
Congenital	4	1.5
Trauma/Post-surgery	163	62.7
Tumor	45	17.3
Infectious	24	9.2
Unknown	24	9.2
Facial palsy stage before facial surgery		
Acute (interval to onset ≤ 4 months)	73	28.1
Chronic (interval to onset > 4 months)	172	66.2
Unknown	15	5.8
Received already a facial therapy before admission		
Yes	154	59.2
No	106	40.8
Facial grading system used prior to surgery		
Yes	139	53.5
No	121	46.5
	**Mean ± SD**	**Median**,** Range**
House-Brackmann scale, pre-surgery	4.3 ± 0.9	4, 2–6
Stennert index, at rest, pre-surgery	2.8 ± 1,4	3, 0–4
Stennert index, in motion, pre-surgery	5.1 ± 1,4	6, 1–6
Interval onset of facial palsy to first surgery, in months	62.0 ± 128,4	8.5, 0-843

SD = standard deviation

### Details on surgery for facial palsy

**Fig. 1:**
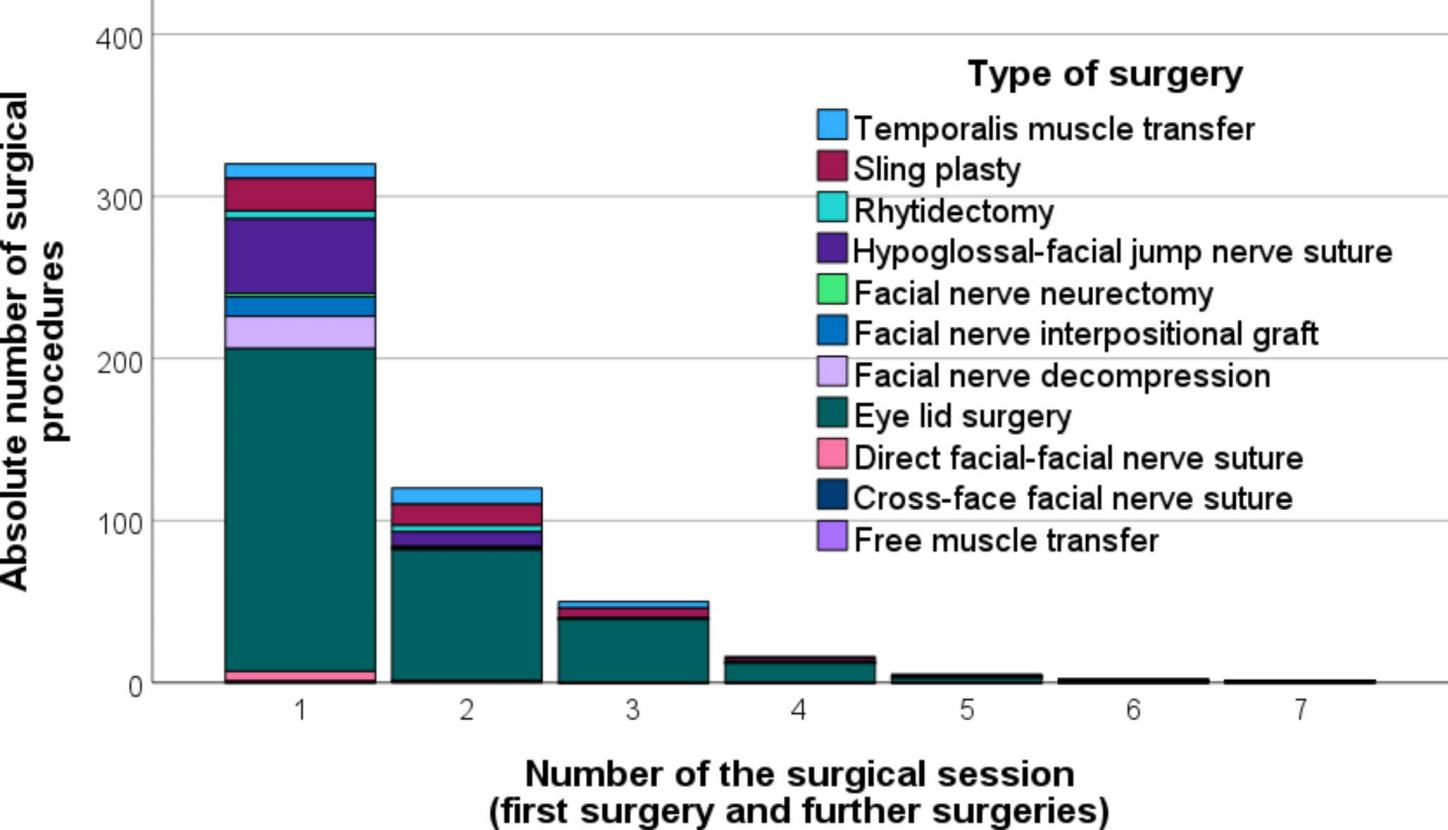
Types of surgical procedures at first facial surgery and at further sessions. Note: The number of procedures is higher than the number of patients

Table 3 gives an overview about all techniques applied during first surgery. There, static procedures were dominating (67.3%), followed by dynamic reconstruction (13.8%), and combined static and dynamic reconstructions (13.5%). The most frequent type of surgery was upper/lower eye lid surgery (76.5%; herein dominating upper lid weight placement [38.5%]), hypoglossal-facial jump nerve suture (17.3%), and facial-facial interpositional graft suture (16.9%). Most patients received one technique at first surgery (73.1%). A combination of two procedures was performed in 17.7%, and of three techniques in 8.5%. The adjuvant treatments are listed in Supplement Table 2). Most frequent were eye protection (57.3%), physiotherapy (30.0%), and drug therapy (25.8%). Figure 1 shows the variety of procedures performed as first surgery and also all following surgeries. Up to seven surgery sessions were counted. Eyelid surgery were also the dominant type of surgery in subsequent surgical sessions.

**Table 3:** Details on first surgery for facial palsy

Parameter	*n*	%
All patients	260	100
Type of reconstruction		
Static reconstruction	175	67.3
Dynamic reconstruction	36	13.8
Combined static and dynamic reconstruction	35	13.5
Other	14	5.4
Type of surgery*		
Upper or lower eye lid surgery	199	76.5
Hypoglossal-facial jump nerve suture	45	17.3
Facial-facial interpositional graft suture	44	16.9
Static sling plasty for the angle of the mouth	21	8.1
Decompression of the facial nerve	20	7.7
Temporalis muscle plasty	9	3.5
Direct facial-facial nerve suture	8	3.1
Facial nerve neurectomy	4	1.5
Rhytidectomy	4	1.5
Cross-face nerve suture	1	0.4
In combination with other non-facial nerve surgery	79	30.4
Number of different surgical procedures		
1	190	73.1
2	46	17.7
3	22	8.5
4	2	0.8
Details on the eye surgery*		
Upper lid weight	100	38.5
Tarsorrhaphy and variations	69	26.5
Lower lid resection	42	16.2
Kanthoplasty	37	14.2
Eye brow lift	22	8.5
Blepharoplasty	16	6.2
Release of a tarsorrhaphy	8	3.1
Upper lid weight explantation	4	1.5
Myectomy of parts of the orbicularis oculi muscle	1	0.4
Number of eye surgery procedures		
0	61	23.5
1	119	45.8
2	61	23.5
3	18	6.9
4	1	0.4
	**Mean ± SD**	**Median**,** Range**
Surgery time, in minutes	102.5 ± 97.4	73.6, 5-853
Upper lid weight, in gram	1.3 ± 0.2	1.2, 0.6–1.8
Duration of inpatient treatment, in days	7.4 ± 12.4	5, 0-160
Overall number of surgeries	1.7 ± 1.0	1, 1–7

*Sum is higher than the number of patients as some patients received several types of reconstruction; SD = standard deviation;

### Complications of surgery

**Fig. 2:**
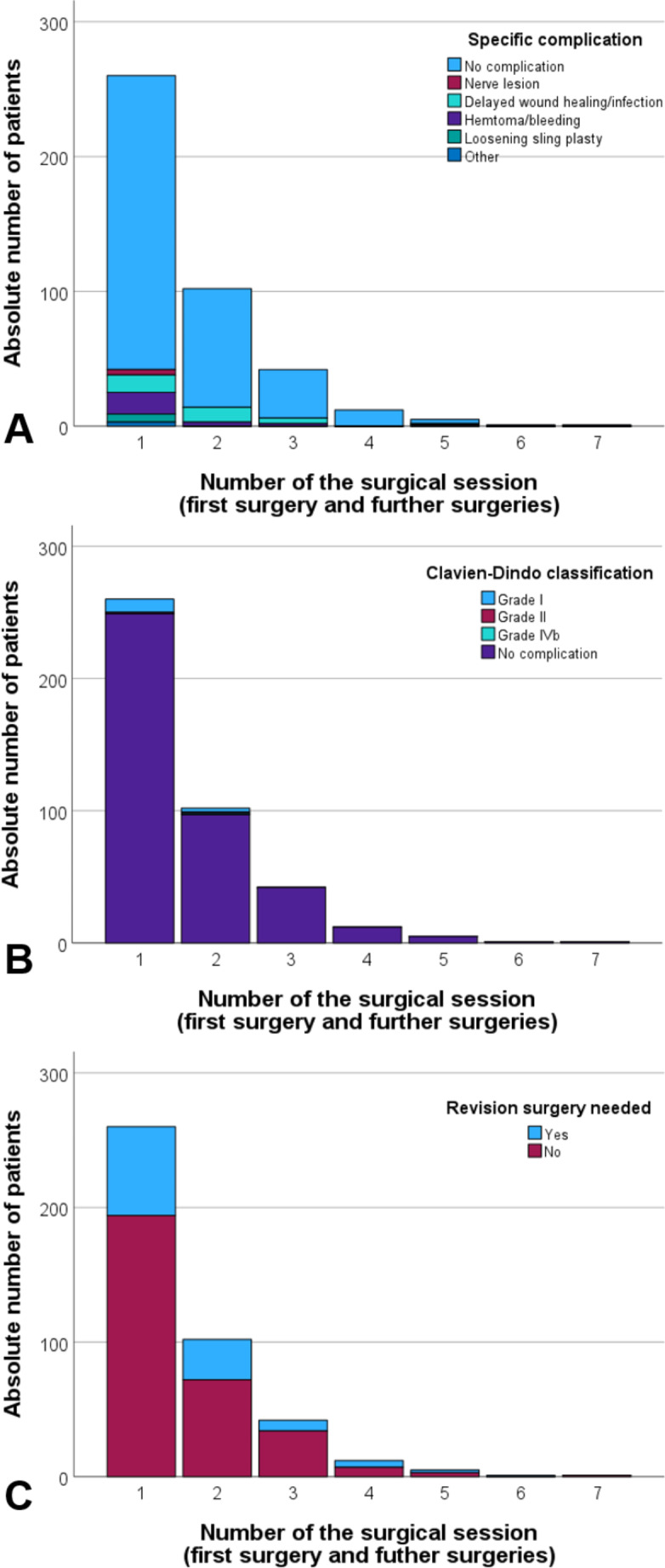
Complications and needed revision surgery at first facial surgery and at further sessions. **A**: Specific complication due to facial surgery. **B**: Clavien-Dindo classification of complications. **C**: Proportion of patients needing revision surgery

The postoperative complications after first facial surgery are summarized in Table 4; Fig. 2. No complications was seen after most surgeries. Bleeding/hematoma formation needing revision surgery was the most frequent but still seldom complication with an occurrence of 6.2%. Other specific complications were very rare and severe complications were not seen. 3.9% and 0.4% of the surgery were followed by a CDC grade I and grade II, respectively. Higher CDC grades were not observed. Most subsequent surgeries also proceeded without complication (cf. Figure 2 and Supplement Table 3). In general, 25.4% needed revision surgery after first surgery, mainly to improve functional outcomes.

**Table 4:** Complication of first surgery for facial palsy

Parameter	*n*	%
All patients	260	100
Clavien-Dindo classification		
No	249	95.8
Grade I	10	38.5
Grade II	1	0.4
Specific complications		
No	218	83.8
Nerve injury	4	1.5
Delayed wound healing/wound infection	13	5.0
Bleeding/hematoma needing revision surgery	16	6.2
Sling plasty loosening	6	2.3
Other	3	1.2
Revision surgery needed		
Yes	66	25.4
No	194	74.6
**Parameter**	**Mean ± SD**	**Median**,** Range**
Follow-up time, months	33.4 ± 42.1	14, 1-202
Number of follow-up visits	2.9 ± 42	0–25

SD = standard deviation

### Patients’ satisfaction with the surgical outcome

**Fig. 3:**
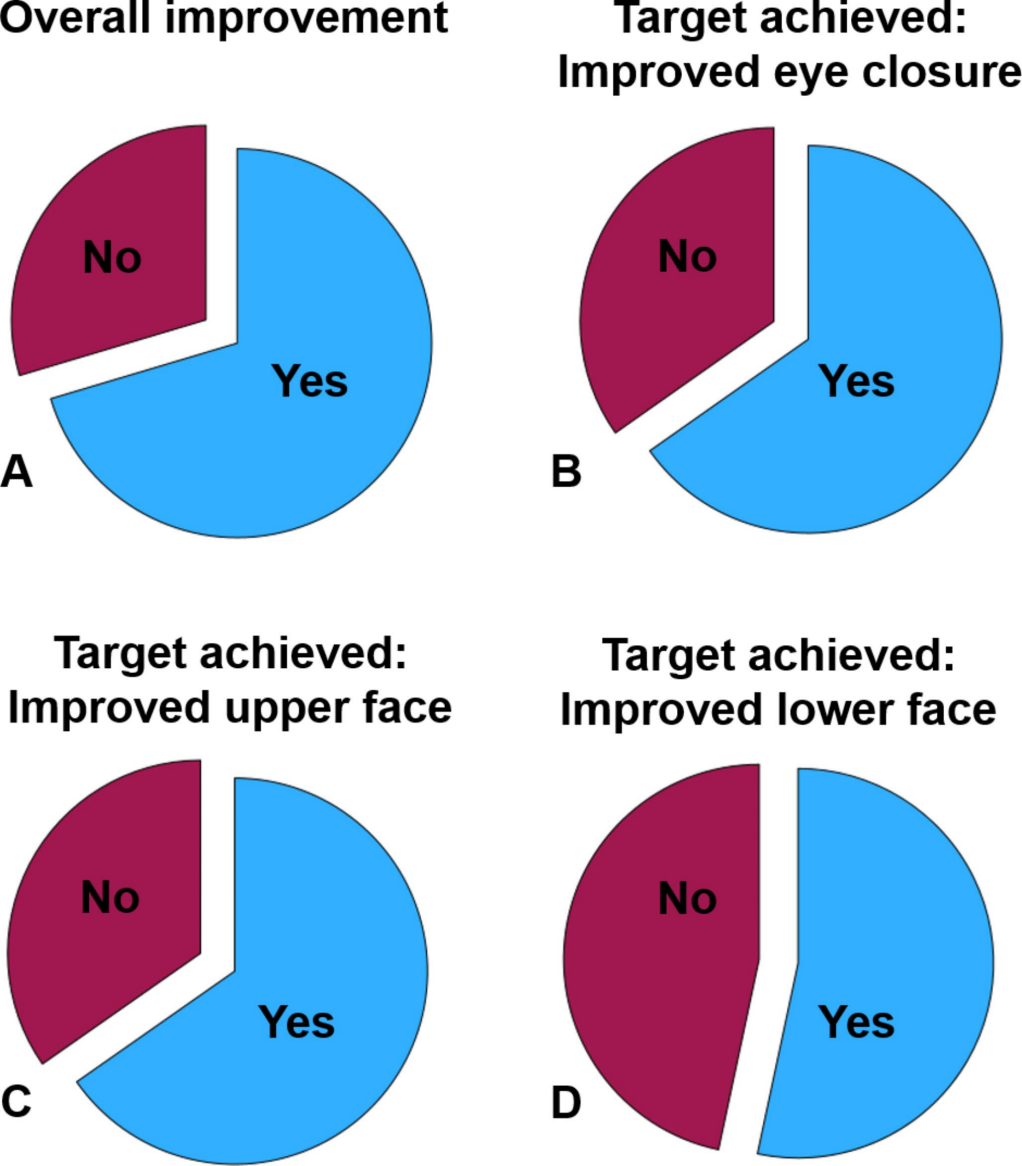
Overall patients’ satisfaction with facial surgery. **A**: Overall improvement. **B**: Improved eye closure. **C**: Improved upper face function. **D**: Improved lower face function

The median time to a measured reinnervation after facial nerve reconstruction was 14 months. Details on patients’ satisfaction with the outcome are listed in Table 5. Overall, 70.4% of the patients were satisfied with the final result. The satisfaction rate was dependent on the target area of the surgery (Fig. 3). The satisfaction was higher if the target of the facial surgery was to improve eye closure (65.2%) or to improve upper face function (65.3%) than if the target was to improve the lower face function (53.3%). Facial grading using the House-Brackmann scale or the Stennert index both at baseline and later on to assess the final outcome was only performed in 10% and 12.5% of the patients, respectively. For these patients, the House-Brackmann scale improved by -0.5 ± 1.1 points from before to after surgery. The Stennert index at rest improved by -0.6 ± 1.2 points. The Stennert index in motion improved by -0.8 ± 1.4 points.

**Table 5:** Patient’s satisfaction with the outcome of surgery and final grading

Parameter	*n*	%
Overall satisfaction with facial function after surgery	260	100
Yes	183	70.4
No	77	29.6
Target: improve eye closure	164	100
Achieved	107	65.2
Not achieved	57	34.8
Target: improve function in upper face	193	100
Achieved	126	65.3
Not achieved	67	34.7
Target: improve function in lower face	135	100
Achieved	72	53.3
Not achieved	63	46.7
House-Brackmann scale, improved after surgery		
Yes	7	2.7
No	19	7.3
Not measured	234	90.0
Stennert index, at rest, improved after surgery		
Yes	21	8.1
No	35	13.5
Not measured	204	78.5
Stennert index, in motion improved after surgery		
Yes	20	7.7
No	36	13.8
Not measured	204	78.5
	**Mean ± SD**	**Median**,** range**
Time to reinnervation in case of facial nerve reconstruction, in days	14.0 ± 3.1	14, 7–13
House-Brackmann scale, final outcome after surgery, *n* = 26	-0.5 ± 1.1	0, -4-1
Stennert index, at rest, final outcome after surgery, *n* = 56	-0.6 ± 1.2	0, -3-4
Stennert index, in motion, final outcome after surgery, *n* = 56	-0.8 ± 1.4	0, -4-3

SD = standard deviation

Several factors were associated with patient’s satisfaction with the outcome of surgery (Table 6). If a facial nerve reconstruction could be performed, the overall satisfaction was higher than if the nerve was not reconstructed (*p* = 0.040). The satisfaction was especially high, when the nerve reconstruction was combined with an upper lid weight (*p* = 0.034). If the target was to achieve an improved eye closure, the satisfaction was higher if an upper lid weight was implanted (*p* = 0.003). The satisfaction was higher if only one surgery was needed compared to the need of later revision surgery (*p* = 0.035). Satisfaction with improvement in the upper face was also higher if no revision surgery was needed (*p* = 0.004). If the target was to improve the lower face, dynamic reconstruction ± static reconstruction achieved better results than static measures alone (*p* = 0.004). Facial nerve reconstruction surgery produced higher satisfaction than no nerve reconstruction surgery (*p* = 0.007), especially in combination with upper lid weight surgery (0.022). The postoperative decrease of the House-Brackmann scale, i.e. increase of facial function, was higher if the patients were satisfied with result (*p* = 0.007), if the target was better eye closure (*p* = 0.003), or better upper face function (*p* = 0.002). A postoperative increase of the Stennert index at rest, i.e. increase of facial function at rest, was higher, if the patient was overall satisfied with the outcome (*p* = 0.001), if the target was better upper face function (*p* = 0.022), or better lower face function (*p* = 0.003). Finally, postoperative increase of the Stennert index in motion, i.e. increase of facial function in motion, was higher, if the patient was overall satisfied with the outcome (*p* < 0.001), or if the target was a better lower face function (*p* = 0.004).

**Table 6:** Factors associated with patient’s satisfaction with the outcome of surgery

Parameter	Overall improvement			Target achieved: Improved eye closure			Target achieved: Improved upper face			Target achieved: Improved lower face		
	Yes	No	*p*	Yes	No	*p*	Yes	No	*p*	Yes	No	*p*
	*n*	*n*		*n*	*n*		*n*	*n*		*n*	*n*	
Gender			0.406			0.070			0.091			0.595
Female	73	35		37	28		48	34		31	30	
Male	110	42		70	29		78	33		41	33	
Charlson Comorbidity Index (CCI)			0.176			0.096			0.390			0.714
CCI = 0–2	107	38		67	28		69	41		47	43	
CCI ≥ 3	76	39		40	29		57	26		25	20	
Etiology trauma/post-surgery			0.444			0.684			0.748			0.267
Yes	71	26		41	20		50	25		22	25	
No	112	51		66	37		76	42		50	38	
Etiology tumor			0.809			0.205			0.750			0.924
Yes	152	63		88	47		102	56		61	53	
No	31	14		19	10		23	11		11	10	
Type of reconstruction			0.237			0.236			0.839			**0.004**
Static reconstruction	117	58		74	34		80	44		26	41	
Dynamic reconstruction	26	10		9	11		19	9		17	11	
Combined static/dynamic reconstruction	28	7		19	10		20	12		21	10	
Other	12	2		5	2		7	2		8	1	
Facial nerve reconstruction surgery			**0.040**			0.255			0.714			**0.007**
Yes	51	13		25	18		37	18		37	18	
No	132	64		82	39		89	49		35	45	
Upper lid weight			0.198			**0.003**			0.377			0.434
Yes	75	25		60	18		50	31		38	29	
No	108	52		47	39		76	36		34	34	
Static sling plasty for angle of the mouth			0.425			0.973			0.442			0.848
Yes	37	19		19	10		19	13		17	14	
No	146	58		88	47		107	54		55	49	
Nerve reconstruction surgery ± lid weight			**0.034**			0.145			0.836			**0.022**
Yes, combined surgery	25	4		17	8		18	10		20	8	
No, nerve surgery alone	26	9		8	10		19	8		17	10	
No, other surgery	132	64		82	39		89	49		35	45	
Clavien-Dindo complication (CDC)			0.616			0.867			0.718			0.124
Yes (CDC ≥ I)	7	4		5	3		6	4		3	7	
No (CDC = 0)	176	73		102	54		120	63		69	56	
Specific facial surgery complication			0.344			0.441			0.159			0.746
Yes	27	15		18	7		15	13		10	10	
No	156	62		89	50		111	54		62	53	
Revision surgery needed			0.444			**0.035**			**0.004**			0.827
Yes	44	22		23	21		26	27		16	15	
No	139	55		84	36		100	40		56	48	
	**Mean ± SD**	**Mean ± SD**		**Mean ± SD**	**Mean ± SD**		**Mean ± SD**	**Mean ± SD**		**Mean ± SD**	**Mean ± SD**	
Age at onset of palsy, years	55.3 ± 20.9	57.1 ± 23.1	0.393	55.8 ± 19.7	53.4 ± 22.8	0.079	56.6 ± 20.6	51.2 ± 21.5	0.598	51.1 ± 19.0	52.0 ± 24.0	0.083
Age at first surgery, years	60.3 ± 18.5	62.7 ± 19.8	0.891	61.2 ± 16.6	58.0 ± 21.5	**0.004**	61.0 ± 18.4	60.0 ± 19.8	0.635	54.0 ± 18.3	57.8 ± 21.0	0.339
Interval onset of facial palsy to first surgery, in months	58.1 ± 117.0	70.5 ± 151.7	0.345	66.8 ± 138.7	49.4 ± 81.9	**0.048**	54.2 ± 110.6	61.2 ± 108.0	0.527	32.6 ± 50.2	80.5 ± 144.5	**< 0.001**
House-Brackmann scale, pre-surgery	4.3 ± 0.9	4.4 ± 0.9	0.929	4.3 ± 0.8	4.3 ± 0.8	0.622	4.3 ± 0.9	4.5 ± 0.0.8	0.430	4.5 ± 0.8	4.2 ± 0.9	0.554
House-Brackmann scale, Change pre-surgery/post-surgery	-0.7 ± 1.3	0.1 ± 0.6	**0.007**	-1.0 ± 1.5	0 ± 0.6	**0.003**	-0.9 ± 1.4	0 ± 0.5	**0.002**	-0.8 ± 1.0	-0.3 ± 1.3	0.830
Stennert index, at rest, pre-surgery	2.8 ± 1.4	2.7 ± 1.2	0.511	3.1 ± 1.4	2.8 ± 1.1	0.720	3.0 ± 1.2	2.6 ± 1.4	**0.034**	2.9 ± 1.3	2.8 ± 1.3	0.622
Stennert index, at rest, Change pre-surgery/post-surgery	-0.7 ± 1.2	0.1 ± 0.6	**0.001**	-0.7 ± 0.12	0.7 ± 1.3	0.836	-1.0 ± 1.2	-0.1 ± 0.9	**0.022**	-1.0 ± 1.3	-0.2 ± 0.83	**0.003**
Stennert index, in motion, pre-surgery	5.1 ± 1.4	5.1 ± 1.0	0.133	5.2 ± 1.4	5.4 ± 1.2	0.437	5.3 ± 1.2	5.1 ± 1.5	0.393	5.3 ± 1.3	5.1 ± 1.3	0.757
Stennert index, in motion, Change pre-surgery/post-surgery	-0.9 ± 1.5	-0.1 ± 0.3	< **0.001**	-0.9 ± 1.5	-0.7 ± 1.1	0.108	-1.1 ± 1.5	-0.6 ± 1.3	0.148	-1.3 ± 1.5	-0.3 ± 1.1	**0.004**

SD = standard deviation

### Surgical rates over the years

The number of habitants in Thuringia varied between 2,108,863 and 2,311,140 habitants from 2006 to 2022 (men: 1,043,936 to 1,139,051; women: 1,064,927 to 1,172,089). The average facial surgery rate was 0.70 ± 0.28/100,000 per year. The average surgery rate was higher for men than for women (men: 0.83 ± 0.39; women 0.58 ± 0.24).

## Discussion

Facial palsy is the most common cranial nerve disease with an overall incidence of 25–55 peripheral facial palsies per 100,000 person years [2, 18] Depending on the etiology, most palsies show a good recovery under drug treatment [2, 19], but in case of severe lesion, about 1–5 per 100.000 person years do not recover or develop a defect healing [9]. These patients are candidates for facial surgery to improve facial function and facial esthetics. Recently, it has been shown that otolaryngology is the dominating discipline to provide inpatient surgery for facial paralysis in Germany [9]. In accordance with this recognition, the presented analysis reflects the importance of the discipline for the surgical treatment of patients with facial paralysis and severe deficits in facial function. The presented population-based study shows facial surgery was mainly indicated for trauma/post-surgery and tumor-related facial paralysis (totaling 80% of the cases). This might explain why upper lid weight implantation was by far the most important technique. Upper lid weight loading is a fast and efficient technique to improve eye closure and can be used for patients with permanent facial paralysis but also for patients with severe acute facial paralysis with unclear prognosis [20]. The etiologies might also explain why hypoglossal-facial jump nerve suture and facial-facial interpositional graft suture were the dominating facial nerve reconstruction techniques. Facial-facial interpositional graft suture is needed to restore facial function in patients with malignant parotid tumor and complex tumor infiltrations of the peripheral facial plexus [21]. With longer denervation time, the chance of a good functional result decreases when using a direct reconstruction technique. Here, or in case of a very proximal lesion, like after vestibular schwannoma surgery, a hypoglossal-facial jump nerve suture is an often chosen technique [22, 23]. Most important, and this is shown to our knowledge for the first time, that this kind of surgery is performed with a very low complication rate and a patient satisfaction rate of about 70%. Even though many patients need further surgeries to refine the results.

The present study has some limitations. Due to the retrospective design, decision making could not be analyzed, i.e. we could not analyze why a certain technique or combinations of techniques and not others were chosen in individual cases. Furthermore, only associations between surgery and outcome could be analyzed but no causalities. It might also be that we underestimated the number of treated inpatient cases as we only selected patients coded for the diagnosis of a facial palsy and related surgery. If only the procedure was coded, but not the underlying disease, it is possible that we have not recorded this case. Furthermore, outpatient procedures were not included. Upper lid loading, for instance, can also be performed as an outpatient procedure. Most important is the limited outcome analysis. In prospective studies or in specialized centers, often standardized facial grading systems like nowadays the Sunnybrook grading, eFACE or others are used to assess facial function [11, 24, 25]. Furthermore, facial-specific patient reported outcome measures (PROMs) like the facial disability index (FDI), the Facial Clinimetric Evaluation (FaCE) scale, or the FACE-Q paralysis module are applied to capture the patient’s perspective [11, 26, 27]. Unfortunately, neither one is standard in clinical routine setting. The present study revealed that such measures were performed at baseline and later on only in a minority of patients.

What is missing are other population-based studies to compare the presented results to other regions. A recent German-wide study analyzing diagnosis-related case group (DRG) coding data of virtually all inpatients who underwent facial surgery for facial palsy between 2005 and 2019 revealed a rather balanced relationship between dynamic nerve reconstruction surgery and static reconstructions (like sling surgery) [9]. Furthermore, the selection of the technique was also dependent on the medical specialty: For instance, lid weights were preferred by otorhinolaryngologists for eye protection, whereas tarsorrhaphy still is the method of choice for most ophthalmologists [9]. The network of Thuringian department of otorhinolaryngology recently performed a population-based study using the same methodology like in the present study to analyze health care of patients with primary parotid cancer and facial paralysis [4]. In this group of patients, also upper lid loading was the important early intervention, and, if feasible, peripheral facial nerve reconstruction. Like in the present study, many patients received further, often minor surgeries, to address specific regional problems (brow plasty, lower lid plasties, slings) [4].

Most important other analyses are hospital-based and are coming from specialized facial nerve centers (for instance [10, 11, 28–31]), . In some centers, free muscle transfer is used for smile reanimation in selected cases [10]. This was so far not the case in Thuringia. Surgery for eye protection plays an important role in all centers (for instance [29]), as well as adjuvant conservative therapy after surgery [10, 29]. Lesions in the cerebellopontine angle are also addressed with direct facial nerve reconstruction [28, 30], whereas such cases would have been treated by a cross-nerve suture like the hypoglossal-nerve jump nerve suture in the present study or by others [11]. The number of reports using masseteric-facial nerve suture is also increasing (for instance [31]), . This technique and also the cross-facial nerve suture did not play a role in Thuringia. It seems that at least the hypoglossal and the masseter transfer produce comparable results [32].

The calculated surgery rates in the present study but also in other studies suggest that there is an underuse of facial surgery for patients with permanent facial paralysis, i.e. only a proportion of these patients receive a surgical therapy or a referred to specialized centers [18, 33]. This is this is unsatisfactory as the benefit in terms of improved facial function and quality of life is clearly proven [11, 23, 34–36].

## Conclusions

This population-based analysis analyzing the entire data of a federal state in Germany made it possible to gain an insight into the day-to-day inpatient care of patients with facial nerve palsy who require facial surgical treatment. Most patients reported that they have benefited from the surgery. There is a lack of an obligatory standardized objective description of the treatment results. The hospitals offer a broad spectrum of surgical techniques for facial reanimation surgery. A clinical guideline would help to standardize preoperative diagnostics, the selection of the optimal surgical techniques and outcome reporting.

## Electronic supplementary material

Below is the link to the electronic supplementary material.

Supplementary Material 1

## Data Availability

The data presented in this study are available on request from the corresponding author.
