# Supplementary material for: Surgery for facial palsy in the hands of otorhinolaryngologists: a population-based study
Source: Eur Arch Otorhinolaryngol. 2024 Oct 23;282(2):1061–73. doi: 10.1007/s00405-024-09044-7 (PMC11805891; doi:10.1007/s00405-024-09044-7)
Supplement: Supplementary file 1 — Supplementary Material 1 [file 405_2024_9044_MOESM1_ESM.docx]

**Supplement Table 1**

| **Supplement Table 1. Symptoms** | | |
| --- | --- | --- |
| **Parameter** | **n** | **%** |
| All patients | 260 | 100 |
| Upper face impaired | 250 | 96.2 |
| Eye closure impaired | 225 | 86.5 |
| Lower face impaired | 196 | 75.4 |
| Brow raising impaired | 140 | 53.8 |
| Ectropium | 115 | 44.2 |
| Tearing impaired | 69 | 26.5 |
| Ulceration of the cornea | 39 | 15.0 |
| Speech impaired | 35 | 13.5 |

**Supplement Table 2**

| **Supplement Table 2. Adjuvant therapy** | | |
| --- | --- | --- |
| **Parameter** | **n** | **%** |
| All patients | 260 | 100 |
| Eye protection | 149 | 57.3 |
| Physiotherapy | 78 | 30.0 |
| Drug therapy | 67 | 25.8 |
| Speech therapy | 51 | 19.6 |
| Electrostimulation | 38 | 14.6 |
| EMG biofeedback training | 25 | 9.6 |
| Botulinum toxin | 23 | 8.8 |
| Ergotherapy | 14 | 5.4 |
| Psychotherapy | 12 | 4.6 |
| Telemedicine facial training | 4 | 1.5 |
| Acupuncture | 3 | 1.2 |

**Supplement Table 3**

| **Supplement Table 3. Complications of all surgeries for facial palsy** | | | | | | | | | | | | | | | | |
| --- | --- | --- | --- | --- | --- | --- | --- | --- | --- | --- | --- | --- | --- | --- | --- | --- |
|  | Surgery 1 | | Surgery 2 | | | Surgery 3 | | Surgery 4 | | Surgery 5 | | Surgery 6 | | Surgery 7 | |  |
|  | **n** | **%** | **n** | **%** | **n** | | **%** | **n** | **%** | **n** | **%** | **n** | **%** | **n** | **%** |  |
| Patients undergoing facial surgery | 260 | 100 | 102 | 100 | 42 | | 100 | 12 | 100 | 5 | 100 | 1 | 100 | 1 | 100 |  |
| Clavien Dindo classification |  |  |  |  |  | |  |  |  |  |  |  |  |  |  |  |
| No | 249 | 95.8 | 97 | 95.1 | 42 | | 100 | 12 | 100 | 5 | 100 | 1 | 100 | 1 | 100 |  |
| Grade I | 10 | 38.5 | 3 | 2.9 |  | |  |  |  |  |  |  |  |  |  |  |
| Grade II | 1 | 0.4 | 1 | 0.1 |  | |  |  |  |  |  |  |  |  |  |  |
| Grade IIIa |  |  |  |  |  | |  |  |  |  |  |  |  |  |  |  |
| Grade IVb |  |  | 1 | 0.1 |  | |  |  |  |  |  |  |  |  |  |  |
| Specific complications |  |  |  |  |  | |  |  |  |  |  |  |  |  |  |  |
| No | 218 | 83.8 | 88 | 86.2 | 36 | | 85.7 | 12 | 100 | 3 | 60.0 | 1 | 100 | 1 | 100 |  |
| Nerve injury | 4 | 1.5 |  |  |  | |  |  |  |  |  |  |  |  |  |  |
| Delayed wound healing/Wound infection | 13 | 5.0 | 11 | 10.8 | 4 | | 9.5 |  |  | 1 | 20.0 |  |  |  |  |  |
| Hematoma needing revision surgery | 16 | 6.2 | 3 | 2.9 | 2 | | 4.8 |  |  | 1 | 20.0 |  |  |  |  |  |
| Sling plasty loosening | 6 | 2.3 |  |  |  | |  |  |  |  |  |  |  |  |  |  |
| Other | 3 | 1.2 |  |  |  | |  |  |  |  |  |  |  |  |  |  |
| Revision surgery needed |  |  |  |  |  | |  |  |  |  |  |  |  |  |  |  |
| Yes | 66 | 25.4 | 30 | 29.4 | 8 | | 19.1 | 5 | 41.7 | 2 | 40.0 | 1 | 100 |  |  |  |
| No | 194 | 74.6 | 72 | 70.6 | 34 | | 80.9 | 7 | 58.3 | 3 | 60.0 |  |  | 1 | 100 |  |

**Supplement Table 4**

| **Supplement Table 4.** Surgical rates per 100,000 population per year and for women and men separately. | | | |
| --- | --- | --- | --- |
| **Year** | **Women** | **Men** | **All** |
| 2006 | 0.17 | 0.18 | 0.17 |
| 2007 | 0.34 | 0.44 | 0.39 |
| 2008 | 0.35 | 0.89 | 0.62 |
| 2009 | 0.70 | 0.81 | 0.76 |
| 2010 | 0.62 | 0.82 | 0.72 |
| 2011 | 0.81 | 1.21 | 1.01 |
| 2012 | 0.54 | 0.75 | 0.65 |
| 2013 | 0.55 | 0.66 | 0.60 |
| 2014 | 0.64 | 0.47 | 0.56 |
| 2015 | 1.00 | 1.02 | 1.01 |
| 2016 | 0.73 | 1.69 | 1.20 |
| 2017 | 0.83 | 1.03 | 0.93 |
| 2018 | 0.46 | 0.28 | 0.37 |
| 2019 | 0.93 | 1.33 | 1.12 |
| 2020 | 0.28 | 0.76 | 0.52 |
| 2021 | 0.47 | 1.15 | 0.81 |
| 2022 | 0.37 | 0.67 | 0.52 |
| Mean±SD | 0.58±0.24 | 0.83±0.39 | 0.70±0.28 |
